# Advances in Graphene-Based Electrode for Triboelectric Nanogenerator

**DOI:** 10.1007/s40820-024-01530-1

**Published:** 2024-09-27

**Authors:** Bin Xie, Yuanhui Guo, Yun Chen, Hao Zhang, Jiawei Xiao, Maoxiang Hou, Huilong Liu, Li Ma, Xin Chen, Chingping Wong

**Affiliations:** 1 State Key Laboratory of Precision Electronic Manufacturing Technology and Equipment, Guangdong University of Technology, Guangzhou, 510006 People’s Republic of China; 2 School of Electromechanical Engineering, Guangdong University of Technology, Guangzhou, 510006 People’s Republic of China; 3 School of Materials Science and Engineering, Georgia Institute of Technology, Atlanta, GA 30332 USA

**Keywords:** Triboelectric nanogenerator, Precision processing, Graphene electrode, Self-powered sensor

## Abstract

With the continuous development of wearable electronics, wireless sensor networks and other micro-electronic devices, there is an increasingly urgent need for miniature, flexible and efficient nanopower generation technology. Triboelectric nanogenerator (TENG) technology can convert small mechanical energy into electricity, which is expected to address this problem. As the core component of TENG, the choice of electrode materials significantly affects its performance. Traditional metal electrode materials often suffer from problems such as durability, which limits the further application of TENG. Graphene, as a novel electrode material, shows excellent prospects for application in TENG owing to its unique structure and excellent electrical properties. This review systematically summarizes the recent research progress and application prospects of TENGs based on graphene electrodes. Various precision processing methods of graphene electrodes are introduced, and the applications of graphene electrode-based TENGs in various scenarios as well as the enhancement of graphene electrodes for TENG performance are discussed. In addition, the future development of graphene electrode-based TENGs is also prospectively discussed, aiming to promote the continuous advancement of graphene electrode-based TENGs.

## Highlights

- Comprehensively reviewed the progress in research on graphene electrode-based triboelectric nanogenerators (TENGs) from two dimensions, including precision processing methods of graphene electrodes and applications of TENGs.
- Discussed the various applications of graphene electrode-based TENGs in different scenarios, as well as the ways in which graphene electrodes enhance the performance of TENGs.
- Offered a prospective discussion on the future development of graphene electrode-based TENGs, with the aim of promoting continuous advancements in this field.

## Graphical abstract

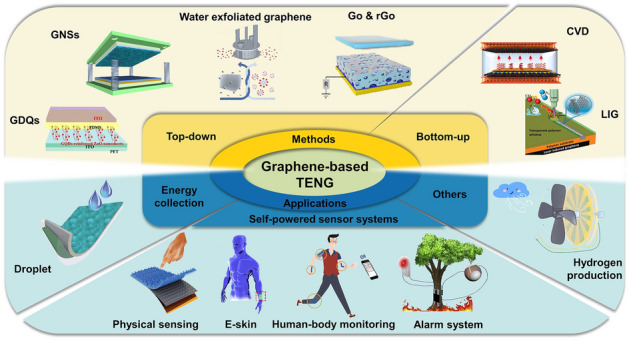

## Introduction

The advent of artificial intelligence (AI) and the Internet of Things (IoT) has led to the widespread application of wearable electronics. The issue of sustainable energy supply for these devices represents a pressing concern that must be addressed in the present era [1–4]. Conventional battery technologies necessitate frequent charging and replacement, thereby contributing to environmental pollution concerns [5]. The emerging triboelectric nanogenerator (TENG) has the potential to convert a wide range of mechanical energy sources [6, 7], including wind [8, 9], wave [10–12], raindrops [13, 14], water mist [15], and human body movement [16, 17] into electricity through the coupled effect of contact charging and electrostatic induction [18–20]. It is therefore anticipated that TENG technology will be able to address the aforementioned issues. Moreover, it provides a reliable energy source for wireless sensor networks, wearable electronic devices, and monitoring systems for health and environmental purposes.

As the key component of TENG, the choice of electrode material plays a critical role in the performance of TENG. Conventional metal electrodes have been identified as being vulnerable to corrosion, and graphene is considered a promising replacement material [21–24]. Due to its excellent electrical conductivity, high transparency, mechanical flexibility, and multifunctionality [25], graphene also shows great potential for applications in piezoelectric nanogenerators [26–28], solar cells [29], microrobots [30], supercapacitors [31, 32], and sensors [33–35]. The studies of Wang et al. and Liu et al. in 2010 demonstrated the ability of graphene to store charge [36, 37], increasing the applicability of graphene in TENG. In 2014 Kim et al. used graphene as an active material in energy-harvesting devices and systems, and fabricated the first transparent flexible graphene-based TENG [25]. Since then, a growing number of graphene-based electrodes for TENG have been developed [14, 38–42].

**Fig. 1:**
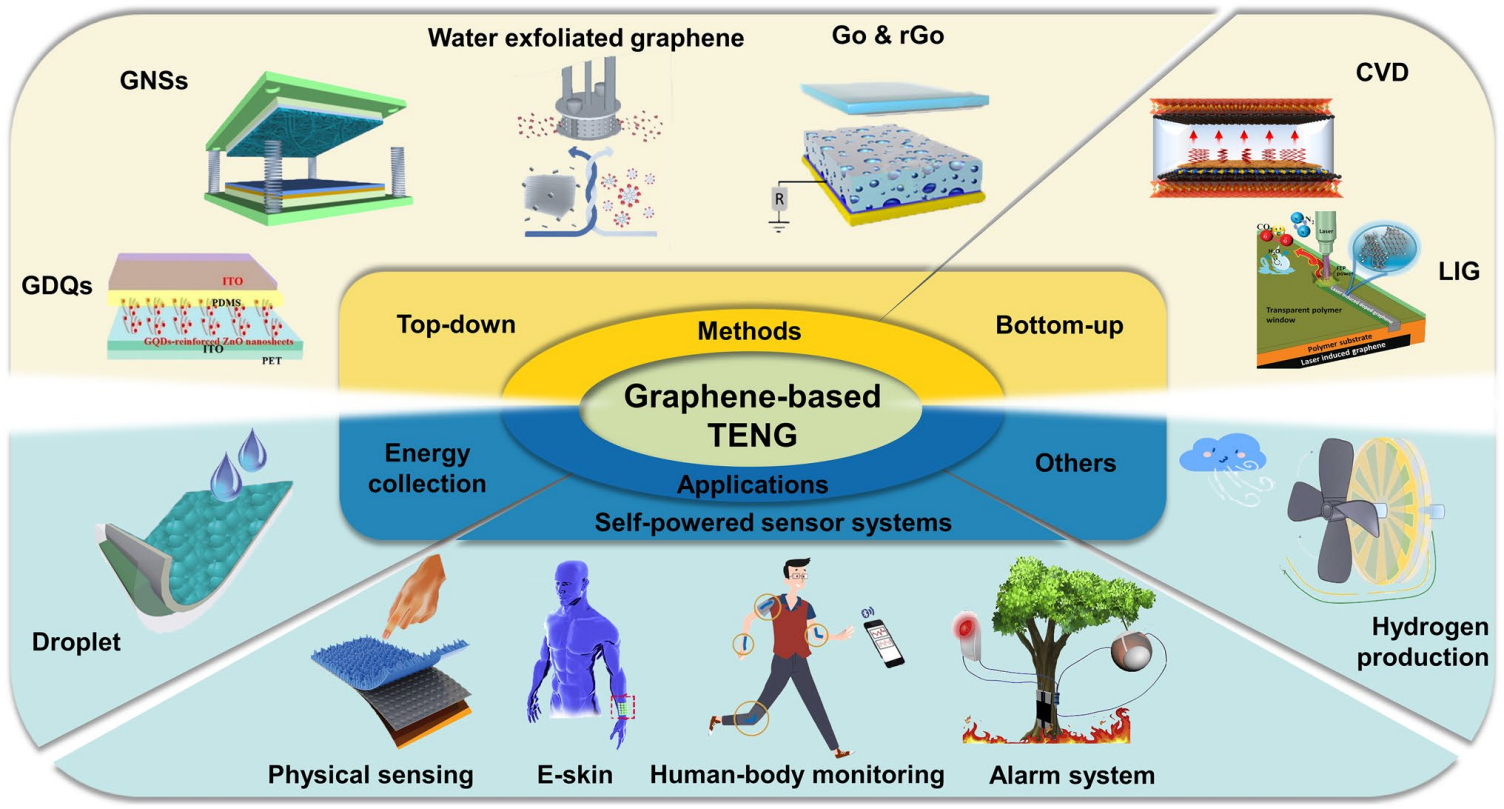
An overview of the latest research progress of graphene electrode-based TENG. GDQs. Reproduced with permission. Reference [43] Copyright 2023, American Chemical Society. GNSs. Reproduced with permission. Reference [44] Copyright 2021, Elsevier. Water-exfoliated graphene. Reproduced with permission. Reference [45] Copyright 2018, Wiley. GO & rGO. Reproduced with permission. Reference [46] Copyright 2018, American Chemical Society. CVD. Reproduced with permission. Reference [47] Copyright 2021, American Chemical Society. LIG. Reproduced with permission. Reference [14] Copyright 2021, Wiley. Droplet. Reproduced with permission. Reference [48] Copyright 2018, Wiley. Physical sensing. Reproduced with permission. Reference [49] Copyright 2022, Wiley. E-skin. Reproduced with permission. Reference [50] Copyright 2020, Elsevier. Human-body monitoring. Reproduced with permission. Reference [51] Copyright 2021, Elsevier. Alarm system. Reproduced with permission. Reference [52] Copyright 2020, Elsevier. Hydrogen production. Reproduced with permission. Reference [53] Copyright 2023, Elsevier

This review examines the research progress of graphene electrode-based TENGs is reviewed from two dimensions, including precision processing methods of graphene electrodes and applications of TENGs. A graphical abstract of this review is shown in Fig. 1. First, multiple precision processing methods for graphene electrodes are described in detail. These methods are classified into top-down and bottom-up processing methods based on the characteristics of the processing methods. Subsequently, the various applications of graphene electrode-based TENGs in energy harvesting, self-powered systems, and other fields are outlined. In addition, the effect of graphene and its derivatives as electrodes on the performance of TENG is discussed. Furthermore, the future directions and potential applications of graphene electrode-based TENG are explored, with the aim of providing valuable insights for the development and innovation in the relevant field.

## Precision Processing Methods for Graphene Electrodes

The excellent electrical and mechanical properties of graphene have led to an increase in demand for this material as an electrode in TENGs. This has resulted in a growing need for precise and efficient processing methods for graphene electrodes. This section provides a summary of the reported precision processing methods for graphene electrodes used in TENGs. As with graphene synthesis methods [54], these can be classified as top-down or bottom-up, depending on the initiating material used.

### Top-Down Methods

The top-down graphene synthesis method employs graphite as the raw material to overcome van der Waals forces between graphene layers, thus enabling the fabrication of single or multiple layers of graphene [55]. Consequently, the processing methods for several TENG electrodes described below are also dependent on graphene precursors and are thus classified as top-down processing methods.

#### Graphene Quantum Dots

Graphene quantum dots (GQDs) are nanoscale carbon-based materials, typically fragments of graphene with dimensions at the nanoscale, featuring quantum size effects and large energy band gaps [56, 57]. This subsection provides on an overview of the functionalization of GQDs by forming composites with other materials and discusses the properties of these GQDs, with a particular focus on their potential for application in electrodes for TENG.

**Fig. 2:**
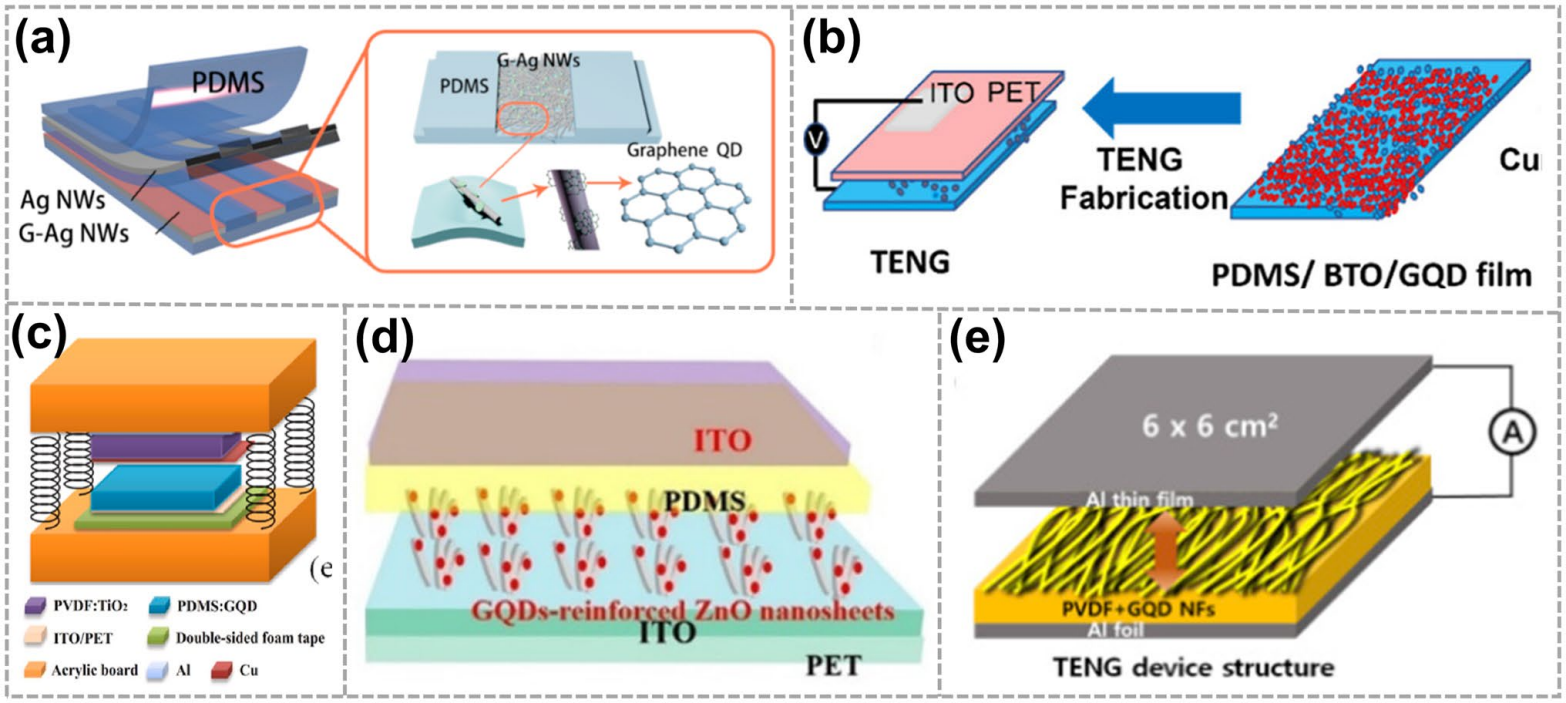
Application of GQDs used as electrodes for TENG. **a** Schematic of the e-skin and the GQDs-coated Ag NW (G-Ag NW) network. Reproduced with permission. Reference [58] Copyright 2018, Elsevier. **b** Schematic of preparation of PDMS/BTO/GQD nanocomposite for TENG devices. Reproduced with permission. Reference [59] Copyright 2024 The Authors. Published by American Chemical Society. **c** Schematic diagram of PT-NG. Reproduced with permission. Reference [60] Copyright 2021, Elsevier. **d** A fabrication process of flexible GQD-reinforced ZnO nanosheet-based FTNG. Reproduced with permission. Reference [43] Copyright 2023, American Chemical Society. **e** Diagram of the TENG device structure. Reproduced with permission. Reference [61] Copyright 2019, Elsevier

As shown in Fig. 2a, Xu et al. employed Ag nanowires (Ag NWs) coated with GQDs as the electrode for triboelectric electronic skin to increase the sensitivity to external mechanical stimulations [58]. For the first time, they modified the Ag NWs with GQDs to improve the electron transfer process between the Ag NWs and polydimethylsiloxane (PDMS). The short-circuit current density is roughly 20 times greater than that of the device without GQDs. Hatta et al. developed a nanocomposite material, comprising PDMS as a polymeric matrix, barium titanite (BTO) nanopowders as dielectric fillers, and GQDs as conductive media (Fig. 2b) [59]. With an increase in GQDs concentration, the nanocomposite films become more crumpled, resulting in increased roughness. This crumpling effect also strengthens the electrostatic and π-π interactions between GQDs and PDMS. Moreover, a higher GQDs concentration facilitates uniform dispersion, creating a network between the polymer matrix, BTO, and GQDs, ultimately enhancing the dielectric properties of the PDMS/BTO/GQDs samples. In a similar vein, Yang et al. fabricated an electrode for piezoelectric-triboelectric nanogenerator (PT-NG) modification by further adding GQDs to the PDMS (Fig. 2c) [60]. The resulting increase in triboelectric output is attributed to the improved dielectric properties of the PDMS film doped with conductive GQDs and the enhanced effective contact caused by the change in PDMS surface microstructure. Moving on, as shown in Fig. 2d, Srivastava et al. first reported the paddy-straw-derived GQDs-reinforced vertical-aligned two-dimensional (2D) ZnO nanosheet-based flexible TENG (FTNG) for scavenging mechanical energy [43]. Choi et al. fabricated polyvinylidene fluoride (PVDF)/GQDs composite nanofibers (NFs), which showed improved TENG performance (Fig. 2e) [61].

In summary, the excellent electrical properties of GQDs enable the effective improvement of dielectric performance in electrodes and the enhancement of output performance in TENG through doping.

#### Graphene Nanosheets

Graphene nanosheets (GNSs) are composed of a blend of monolayer graphene, multilayer graphene (2–10 layers), and nanostructured graphite. In other words, GNSs are a mixture of graphite and graphene, encompassing all types of 2D graphitic materials from those as thin as 0.34 nm to those as thick as 100 nm [62]. GNSs serve as exceptional nanofillers because of their great mechanical, electrical, and thermal properties [63]. It can be incorporated into composite materials to fabricate the precision electrode of TENGs with low interface resistance.

**Fig. 3:**
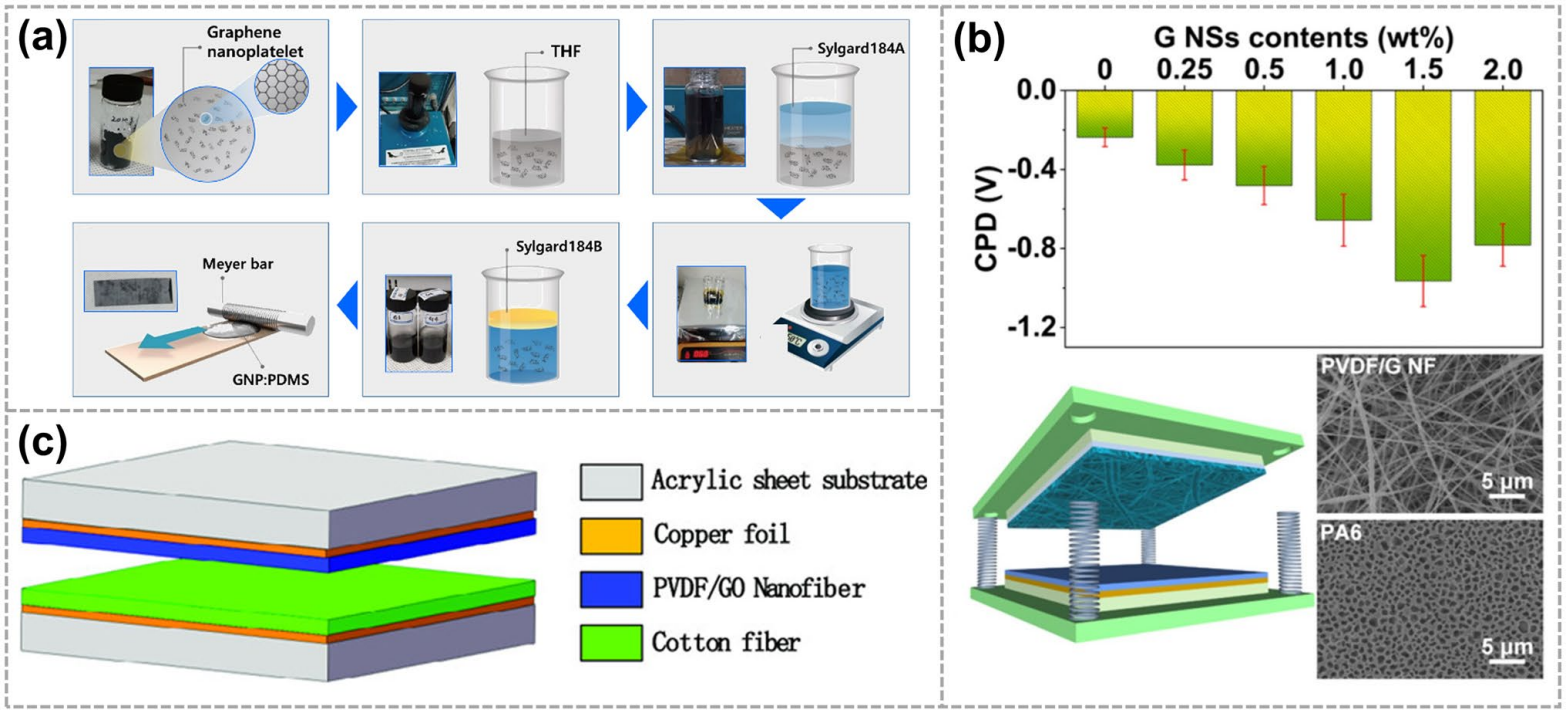
GNSs as fillers for electrodes. **a** Synthesis process of PDMS: GNSs composite material used in an RL-TENG. Reproduced with permission. Reference [64] Copyright 2021, Elsevier. **b** PVDF- GNSs composite nanofibers for fabricating high-performance TENGs. Reproduced with permission. Reference [44] Copyright 2020, Elsevier.** c** 3D structure of the TENG. Reproduced with permission. Reference [65] Copyright 2023, Wiley

Irfan et al. developed a GNSs-filled PDMS matrix that could be used in a rolling-mode TENG (RL-TENG). GNSs were added to increase and optimize the electrical output by improving the dielectric constant of the PDMS friction layer (Fig. 3a) [64]. Similarly, as shown in Fig. 3c, Yang et al. prepared a vertical contact-separated TENG composed of polyvinylidene fluoride (PVDF) and graphene oxide (GO) nanosheets. The electrospinning process used in preparing the composite nanofiber films provides a large effective area, augmenting the output of TENG [65]. In another approach, Lin et al. proposed the construction of high-performance TENGs based on PVDF. They achieved this by incorporating GNSs into PVDF using electrospinning technology. The results demonstrated that both composition modulation with graphene and nanofiber structure fabricated through electrospinning contribute to the triboelectric performance enhancement of PVDF/G NF films (Fig. 3b) [44].

In general, GNSs are typically employed as nanofillers to functionalize elastic polymers, such as PDMS and PVDF. This approach can effectively improve the dielectric constant of the friction layer and enhance the friction electrical properties, thereby improving the output performance of TENG.

#### Water-Exfoliated Graphene

The exfoliation of graphene in water is a common method for the preparation of high-quality graphene films [66], which can be utilized in energy harvesting through TENG. The integration of these films with a variety of wearable and flexible technologies offers new possibilities for the application of TENG in multiple fields.

**Fig. 4:**
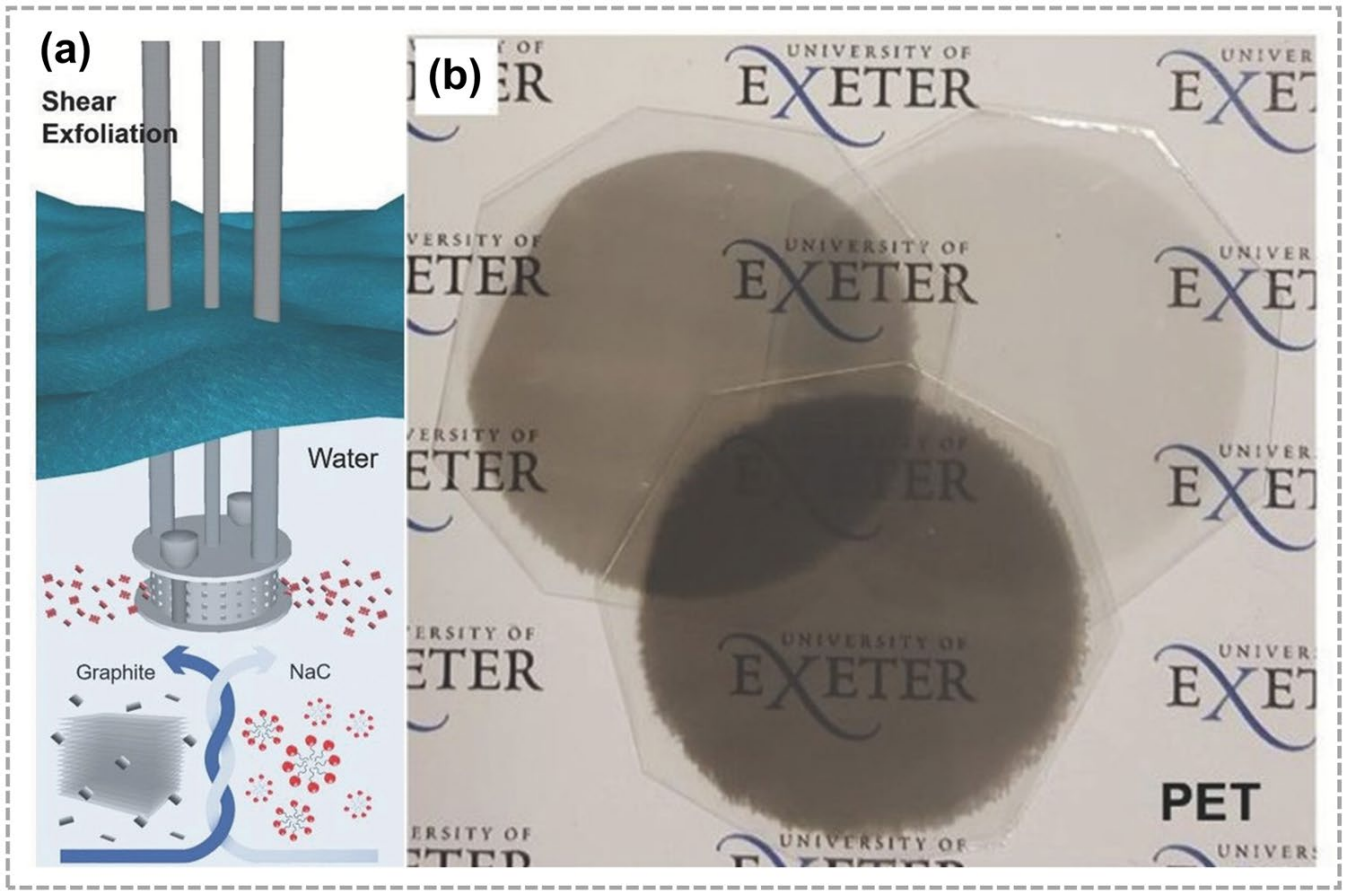
Shear exfoliation of graphite in water and IDT method. **a** Schematic of shear exfoliation of graphite flakes in water. **b** Membrane is detached from the graphene/sub, leaving the SEG film on PET. Reproduced with permission. Reference [45] Copyright 2018, Wiley

As shown in Fig. 4a, Monica et al. proposed a novel method for assembling shear exfoliated graphene (SEG) in water. They demonstrated that SEG flakes suspended in a solution could be conveniently transferred onto various target substrates using the inter-digital transducer (IDT) technique for further applications. Additionally, the researchers combined a single SEG electrode with PDMS to create an active layer. This innovative combination resulted in the development of a flexible and semi-transparent TENG [45]. And Ismael et al. presented TENGs using shear exfoliated graphene as electrodes as well as an active triboelectric layer deposited by a simple solution process. Graphene in combination with polymers such as PDMS was used to produce TENG devices using low-cost solution processing methods, which can be 40 times higher when compared to devices made with aluminum and PDMS [67].

Based on water exfoliation, graphene can be readily deposited on various substrates, serving as electrodes for flexible TENG. This approach can be integrated with a range of wearable applications, thereby opening up new avenues for the utilization of TENG in a multitude of fields.

#### GO & rGO

Graphene oxide (GO) is a chemically modified graphene, which is enriched with negative charges because it is decorated with oxygen functional groups both at the carbon network base planes and at the edges. Consequently, it is an appropriate material for use as a negative triboelectric material in the construction of electrodes for TENG [46, 68].

**Fig. 5:**
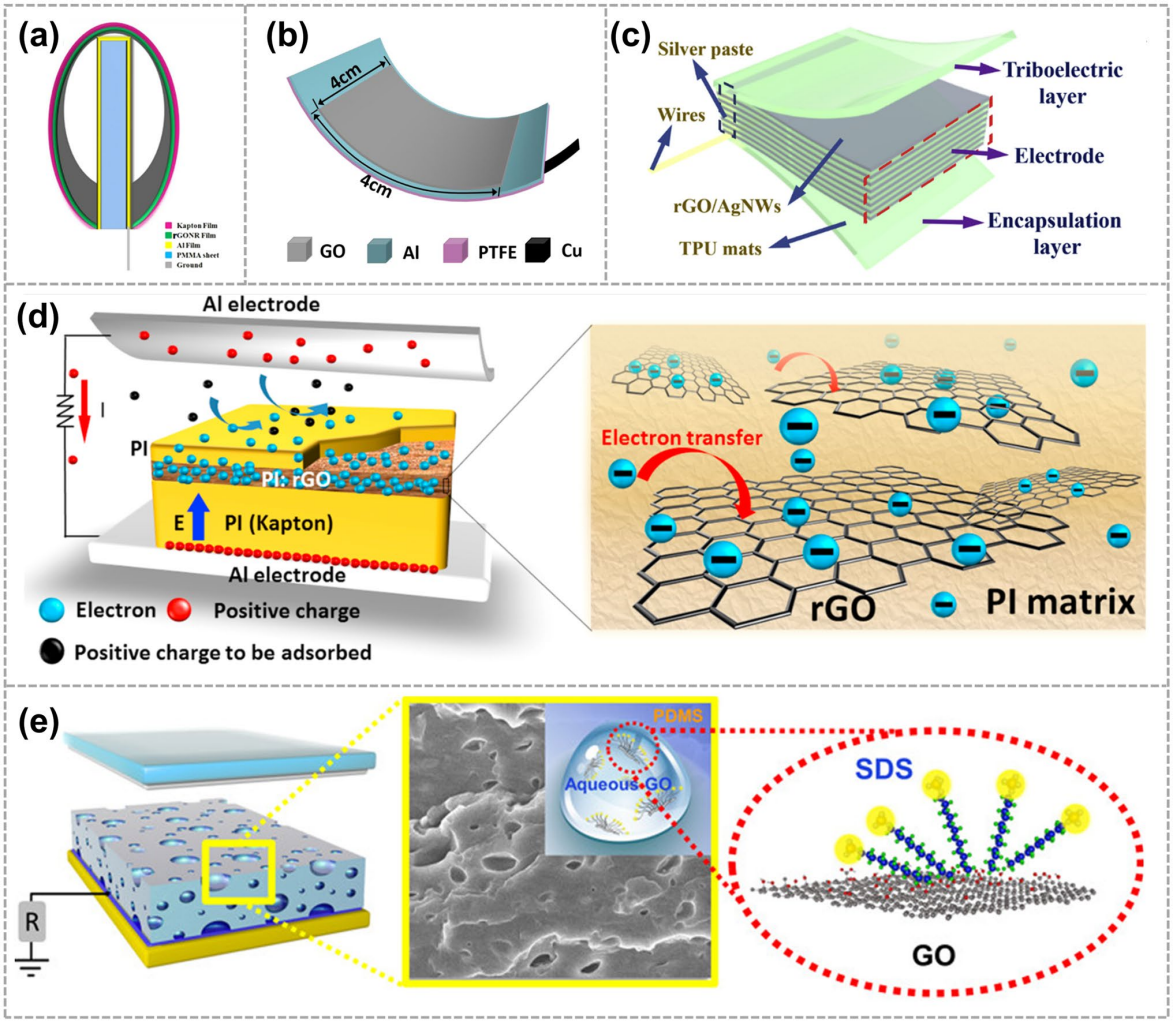
GO and rGO were used as electrodes for TENG. **a** Schematic view of the regions/PVDF-based TENG. Reproduced with permission. Reference [69] Copyright 2016, Springer Nature. **b** Schematic diagram of the GO-enhanced flexible single-electrode TENG. Reproduced with permission. Reference [70] Copyright 2017, American Chemical Society. **c** Schematic structure of our STENG-based e-skin. Reproduced with permission. Reference [50] Copyright 2020, Elsevier. **d** rGO acts as an electron-trapping sites in the friction layer for giant triboelectric enhancement. Reproduced with permission. Reference [71] Copyright 2017, Elsevier. **e** Enhanced power output of a TENG using a modification of PDMS with GO and SDS. Reproduced with permission. Reference [46] Copyright 2018, American Chemical Society

In 2016, Navjot et al. fabricated arch-shaped single electrode-based TENG using a thin film of reduced graphene oxide nanoribbons (rGONRs). As shown in Fig. 5a, the incorporation of rGONRs in PVDF polymer enhances the average surface roughness of rGONRs/PVDF thin film, which improves the charge storage capacity of prepared film [69]. The following year, Guo et al. reported a GO-based single-electrode TENG (S-TENG). By utilizing the ultrathin flexible GO films, the as-fabricated S-TENGs present not only much higher energy harvesting efficiency but also great mechanical durability and low weight (Fig. 5b) [70]. And the same year, Wu et al. enhanced the output performance of the TENGs (Fig. 5d) by introducing reduced graphene oxide (rGO) in the friction layer. The maximum output power density of a vertical contact-separation mode TENG with rGO sheets is 30 times larger than that of a device without rGO sheets [71]. Additionally, in 2018, Viyada et al. introduced a novel method to enhance the properties of PDMS as a negative triboelectric material. They achieved this by incorporating GO and a sodium dodecyl sulfate (SDS) surfactant into the PDMS matrix. The modification of PDMS with GO and SDS resulted in improved performance in the TENG, which is shown in Fig. 5e [46]. In 2020, Zhou et al. fabricated a flexible and self-powered electronic skin (e-skin) based TENG (STENG). The rGO was used to construct a synergic conductive network with AgNWs to prepare stretchable electrodes for the STENG-based e-skin. the e-skin possesses a high sensitivity (78.4 kPa^−1^) as well as a fast response time (1.4 ms) toward pressure, exhibiting the potential prospect of a high-performance tactile sensor (Fig. 5c) [50].

In summary, GO and rGO are significant in the fabrication of precision electrodes for TENG devices. They can improve the power density and charge storage capacity of the electrodes, thereby enhancing the output performance of TENGs. These improvements hold great promise for applications such as enhancing energy harvesting efficiency, improving mechanical durability and sensitivity, and achieving high-performance tactile sensors.

### Bottom-Up Methods

Bottom-up graphene synthesis methods utilize a variety of carbon-containing precursors as feedstock, which are then subjected to a high-energy process of breakdown and conversion into graphene [72]. In other words, these methods begin with smaller carbon-based precursor materials and incrementally build up layer-by-layer to produce graphene through a controlled process. The two approaches outlined below for fabricating graphene electrodes for TENG fall under the classification of bottom-up methods due to their adherence to these characteristics.

#### Chemical Vapor Deposition

Chemical vapor deposition (CVD) is a widely utilized technique for the manufacture of single-layer graphene on transition metal substrates [73]. The graphene produced by this method is of a high quality, with the film thickness and properties being able to be precisely controlled. This makes it a crucial aspect of the manufacturing process of graphene electrodes for TENG.

**Fig. 6:**
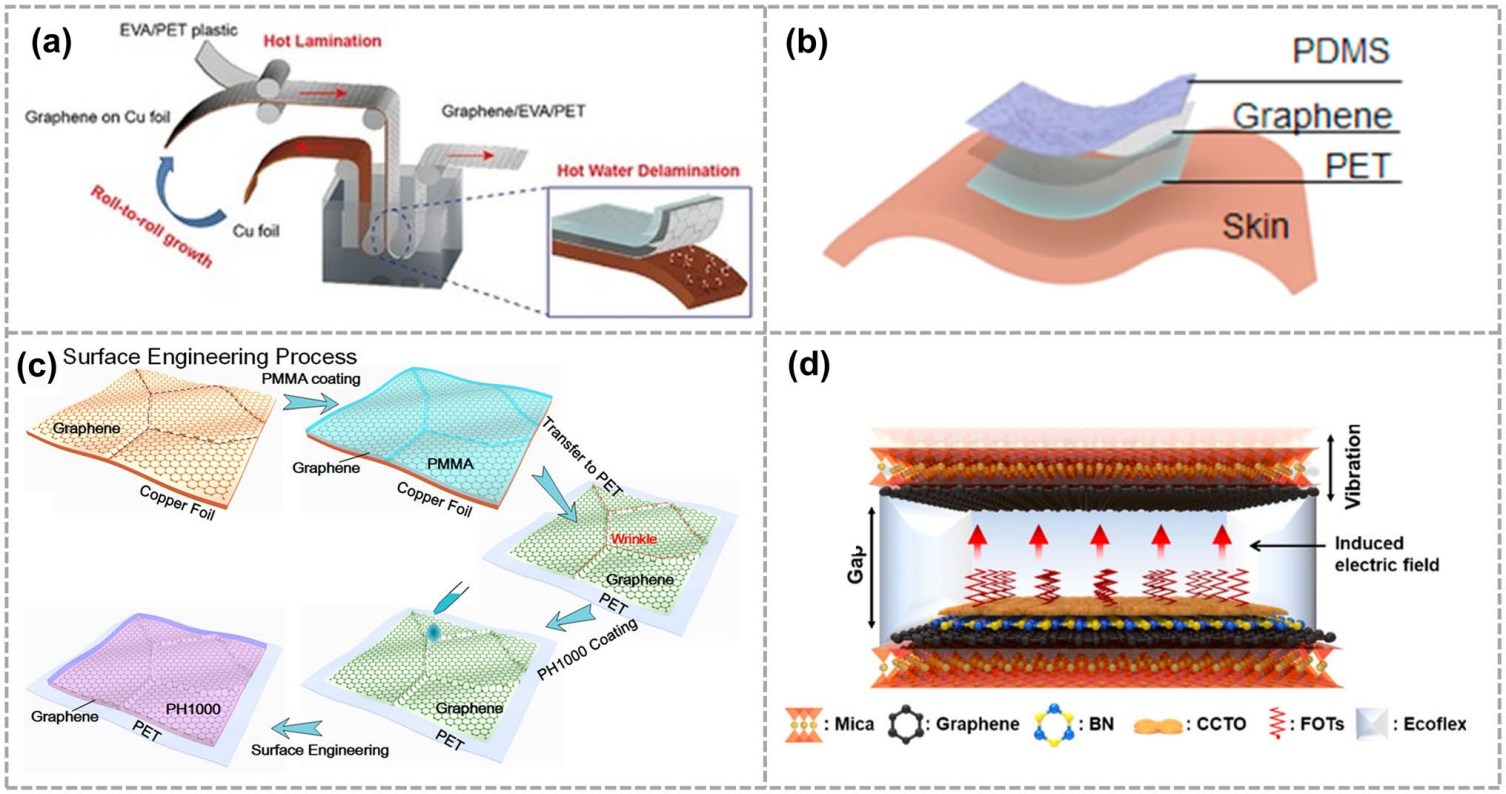
CVD-grown graphene is used for the electrode of the TENG. **a** Schematic illustration of the roll-to-roll delamination of copper and graphene onto EVA/PET substrate. Reproduced with permission. Reference [38] Copyright 2015, Wiley. **b** Structure and fabrication process of CVD-grown graphene on PET/EVA. Reproduced with permission. Reference [74] Copyright 2016, Elsevier. **c** Schematic diagram of GF-based TENG. Reproduced with permission. Reference [75] Copyright 2017, American Chemical Society. **d** Structure of an nc-TENG. Reproduced with permission. Reference [47] Copyright 2021, American Chemical Society

In 2015, Bananakere et al. demonstrated a flexible and transparent TENG device based on graphene/EVA/PET electrodes. They proposed a roll-to-roll "green" method to transfer large-area graphene grown on copper foil by CVD onto flexible, transparent ethylene vinyl acetate/poly (ethylene terephthalate) (EVA/PET) plastic substrates (Fig. 6a) [38]. In the same year, Smitha et al. presented a graphene-based TENG with a CVD grown graphene as the friction layer as shown in Fig. 6b. This choice of graphene as the friction layer provides the TENG with electrical conductivity and high optical transmittance [74]. Additionally, in 2016, Hyenwoo et al. reported a conformal TENG with a graphene electrode. The graphene was grown on Cu foil by the thermal CVD method. The atomically thin thickness of graphene (< 1 nm) enabled the TENG to have low flexural rigidity, facilitating conformal contact on human skin [39]. As shown in Fig. 6c, Yang et al. proposed a high-performance transparent and flexible TENGs based on CVD-grown graphene composite electrodes via surface engineering. And the output current density were enhanced by 140% to 2.4 μA cm^−2^ [75]. Lastly, in 2021, Han et al. proposed a noncontact TENG (nc-TENG). The high-quality graphene grown through CVD served as the electrode of the nc-TENG (Fig. 6d). By combining the optimization of hexagonal boron nitride (h-BN) and 1H,1H,2H,2H-perfluorooctyltrichlorosilane, they achieved an ultra-thin, stable, and high-output performance nc-TENG [47].

The aforementioned studies underscore the potential of graphene in TENGs, which is produced through the CVD technique. The distinctive properties of graphene, including high electrical conductivity, mechanical flexibility, and transparency, render it an optimal material for enhancing the efficiency and functionality of TENGs. The incorporation of graphene into TENGs has led to the development of devices exhibiting enhanced performance, flexibility, transparency, and conformal contact, opening up a range of potential applications.

#### Laser-Induced Graphene

Laser-induced graphene (LIG) is a technology that directly converts polymers into graphene through laser irradiation. This method has attracted considerable interest due to its capacity to be fabricated directly onto a variety of carbon materials using a straightforward process [76, 77]. The favorable electrical conductivity, porous structure, and dopant modulation of LIG hold substantial promise in the realm of flexible wearable electronics and TENG applications [78, 79].

**Fig. 7:**
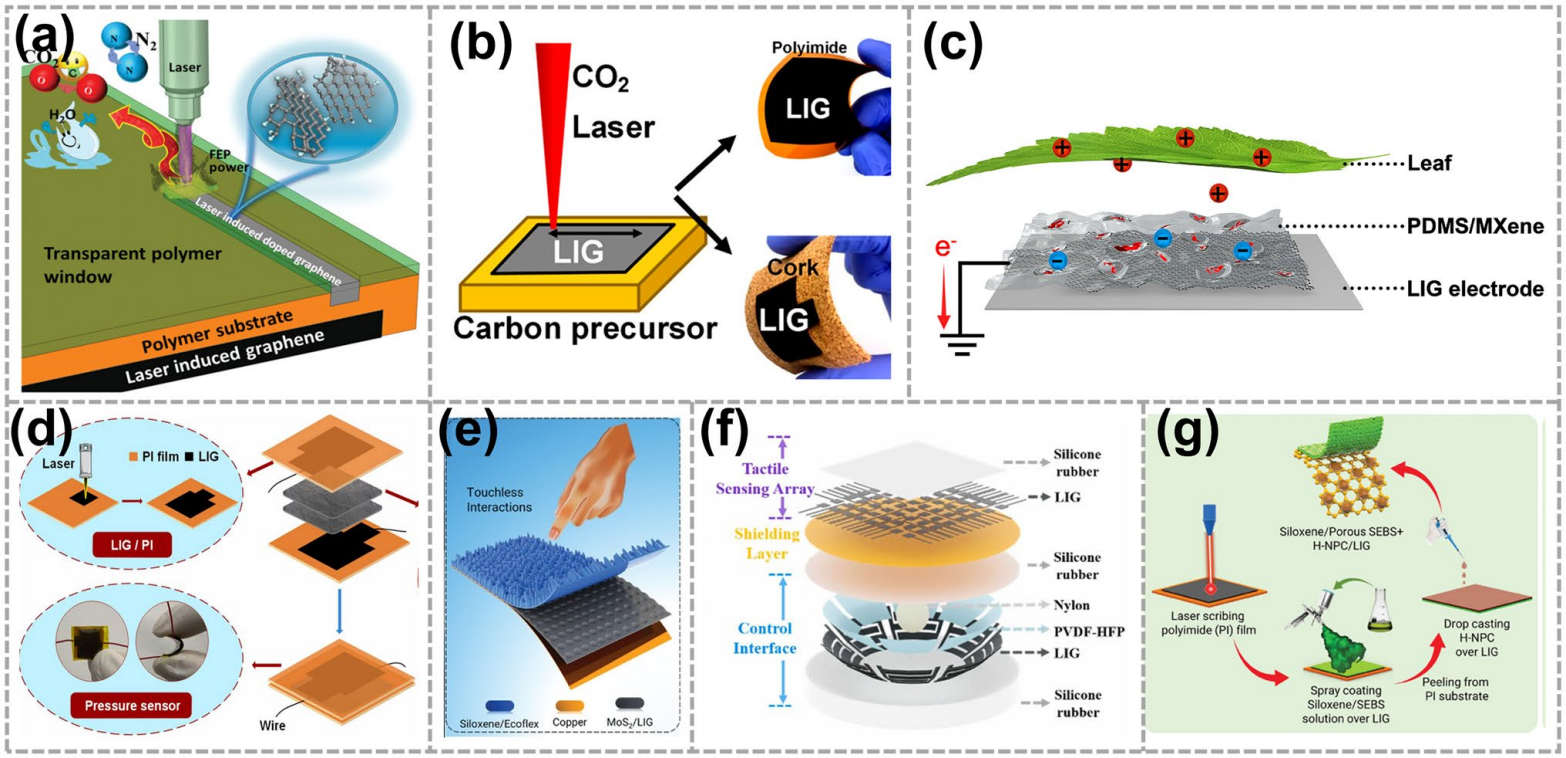
LIG is used as an electrode in TENG. **a** Schematic diagram of interfacial laser-induced F-LIG. Reproduced with permission. Reference [14] Copyright 2021, Wiley. **b** Schematic illustration of the LIG-based TENG. Reproduced with permission. Reference [80] Copyright 2019, American Chemical Society. **c** Working principle in the leaf swing energy collection. Reproduced with permission. Reference [81] Copyright 2019, Elsevier. **d** Schematic diagrams and photos of rGO-cloth/LIG pressure sensor. Reproduced with permission. Reference [82] Copyright 2012, Elsevier. **e** Schematic illustration of contactless TENG. Reproduced with permission. Reference [49] Copyright 2022, Wiley. **f** Schematic representation of the device. Reproduced with permission. Reference [83] Copyright 2023, Elsevier. **g** Schematic diagrams and photos of rGO-cloth/LIG pressure sensor. Reproduced with permission. Reference [84] Copyright 2023, Wiley

In 2021, Chen et al. demonstrated an in situ growing LIG process that enables to pattern of superhydrophobic fluorine-doped graphene on fluorinated ethylene propylene (FEP)-coated polyimide (PI). This method leverages distinct spectral responses of FEP and PI during laser excitation to generate the environment preferentially for LIG formation, eliminating the need for multistep processes and specific atmospheres. The structured and water-repellant structures rendered by the spectral-tuned interfacial LIG process are suitable as electrodes for the construction of a flexible droplet-based electricity generator (DEG) (Fig. 7a) [14]. In 2019, Michael et al. showcased various architectural designs for TENGs utilizing LIG composites (Fig. 7b), which exhibited a high-power output of 0.76 W m^–2^, pioneering the field of LIG-based TENGs [80]. And the same year, Jiang et al. fabricated a highly flexible and effective TENG based on MXene and PDMS composite PDMS/MXene film and LIG electrode. As shown in Fig. 7c, the developed TENG not only realizes effective harvesting of leaf swing energy and human writing energy but also can be utilized to recognize a real-time trajectory. This flexible TENG has enormous potential in the fields of energy harvesting and sensing [81]. Next year, Xia et. al presented a LIG-based pressure sensor (Fig. 7d). The LIG not only contributes to the sensitivity of pressure sensors but also improves the transfer charge density of TENG [82]. As shown in Fig. 7e, Kumar et al. proposed a Siloxene/Ecoflex nanocomposite-based high-performance contactless TENG also in 2022. The introduction of LIG as a charge-trapping intercalation layer increases the surface potential by four-fold, resulting in improved output performance of the TENG [49]. In 2023, Guo et al. developed one device integrated by two different working-mode laser-induced graphene (LIG)-based TENG (Fig. 7f). Thanks to the high-precision machining of LIG, the device can achieve simultaneously its accurate wireless control and tactile pattern recognition capability [83]. And the same year, Kumar et al. designed a breathable triboelectric sensor (Fig. 7g). The LIG as a charge-collecting electrode was successfully transferred to the porous substrate, which was conformally attached to the substrate. This reduces interfacial resistance and minimizes the redundancy of using complex manufacturing techniques [84].

In summary, LIG has demonstrated its versatility with a multitude of applications and notable advantages. Devices based on LIG exhibit flexibility, high-precision control, and the ability to perform a variety of functions. The method offers diverse material options, environmental friendliness, and customizable patterns. Among the precision processing methods previously mentioned, LIG technology is considered the most scalable. Furthermore, LIG films can function as energy harvesting systems, sensors, and contactless devices. When used as an electrode in TENG, LIG can enhance sensitivity, transfer charge density, and output performance, rendering it a promising material for a wide range of technological advancements.

## Applications of Graphene Electrode-Based TENG

This section will present the applications of graphene electrode-based TENG in various fields. In order to gain a comprehensive understanding of the diverse applications of graphene electrode-based TENG, it is necessary to examine several key aspects, including energy harvesting, self-powered sensor systems, the enhancement of TENG performance, and other potential applications.

### Energy Collection

In terms of energy collection, while the ability to collect energy is a common feature of all TENGs, the excellent electrical conductivity and mechanical strength of graphene electrodes, as well as the ability to be doped, offer greater potential for energy collection in TENGs. Consequently, the distinctive properties and advantages of graphene are exploited in a variety of contexts to enhance the efficiency and dependability of energy harvesting capabilities through meticulous design and optimization, thereby maximizing the potential for energy collection in TENGs.

**Fig. 8:**
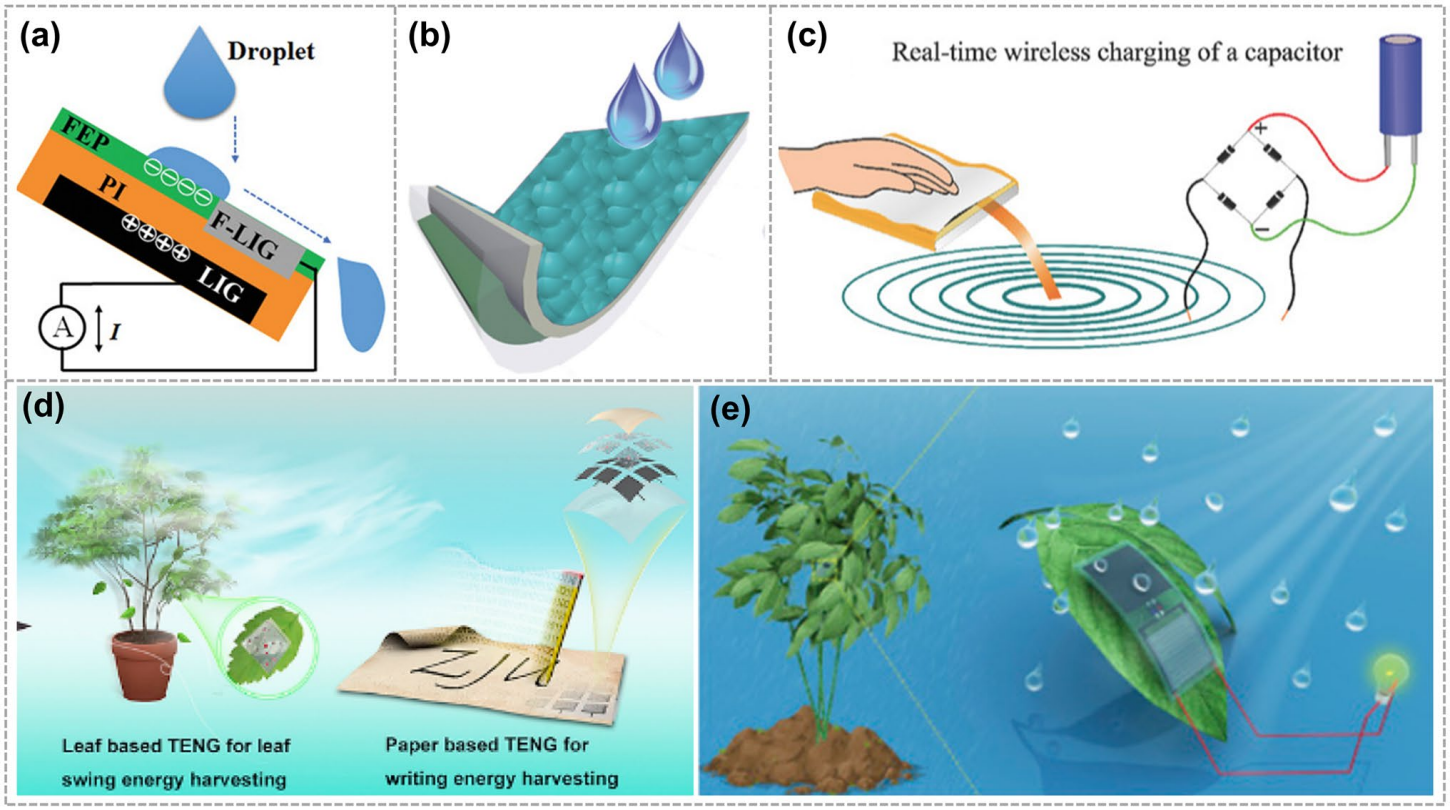
Graphene electrode-based TENG is used for energy collection. **a** Working mechanism of F-LIG-based DEG. Reproduced with permission. Reference [14] Copyright 2021, Wiley. **b** Schematic diagram of the SPS. Reproduced with permission. Reference [48] Copyright 2018, Wiley. **c** A schematic for wireless charging of a capacitor. Reproduced with permission. Reference [85] Copyright 2017, Wiley. **d** Schematic diagram of a self-charging device with bionic self-cleaning interface. Reproduced with permission. Reference [81] Copyright 2019, Elsevier. **e** TENG realizes effective harvesting of leaf swing and human writing energy and is used as a self-driven sensor array to recognize a real-time trajectory. Reproduced with permission. Reference [86] Copyright 2020, Elsevier

In 2021, Chen et al. developed a raindrop energy collecting TENG based on F-LIG, shown in Fig. 8a. The TENG exhibited a peak power density of 47.5 W m^−2^ when a water droplet (105 µL) was released from a height of 25 cm. This device could light up 480 LEDs, showcasing a greatly expanded energy harvesting range [14]. In 2018, Zhang et al. reported a rGo-based self-cleaning/charging power system (SPS), which can be exploited to convert and store energy from falling raindrops directly to provide a stable and durable output. The device can light a light-emitting diode for more than 300 s (Fig. 8b) [48]. Additionally, in the same year, Sai et al. reported a novel additively manufactured gPLA nanocomposite-based high-performance TENG (Fig. 8c). The W-TENG generates very high output voltages > 2 kV with a strong electric field that enables the wireless transmission of harvested energy over 3 m [85]. In 2019, Jiang et al. developed a highly flexible MXene-enabled porous film, which was then integrated with LIG electrode to fabricate TENG. This high-performance MXene-enabled TENG presents versatile applications in harvesting energy from leaf swing and human writing (Fig. 8d) [81]. Furthermore, in 2020, Li et al. developed an integrated self-cleaning and self-charging device based on LIG. The device is capable of harvesting energy from the swinging crop leaves and raindrops. The lotus leaf-like structure enhances the self-cleaning ability of the device and self-cleaning ability allows this device to work reliably in a humid environment (Fig. 8e) [86].

The graphene electrode-based TENG has demonstrated to possess a number of advantages in the field of energy harvesting. It is capable of converting and storing energy from a variety of sources including raindrops, oscillating objects, and human movement. The device can achieve high power density, generate high output performance, and have the capability of wirelessly transmitting the collected energy. Additionally, the devices can self-clean and operate reliably in various environments. These characteristics render the graphene electrode-based TENG a versatile and efficient energy-harvesting solution.

### Self-Powered Sensor Systems

The exponential growth of AI and IoT has led to a surge in demand for sensors, with the issue of their power supply becoming increasingly pressing. One potential solution to this problem is the development of self-powered sensors that obtain energy from the surrounding environment to power themselves [87]. TENG can be effectively used as a novel self-powered sensing technology, which is extensively employed in human body monitoring, alarm systems, electronic skin and other applications in daily life.

#### Physical Sensing Technologies

This section will focus on exploring the practical applications of TENG in sensing physical quantities such as pressure, vibration, strain, and humidity [88–91]. Building upon the exploration of practical applications of TENG in sensing physical quantities, it is evident that innovative advancements have been made in various fields.

**Fig. 9:**
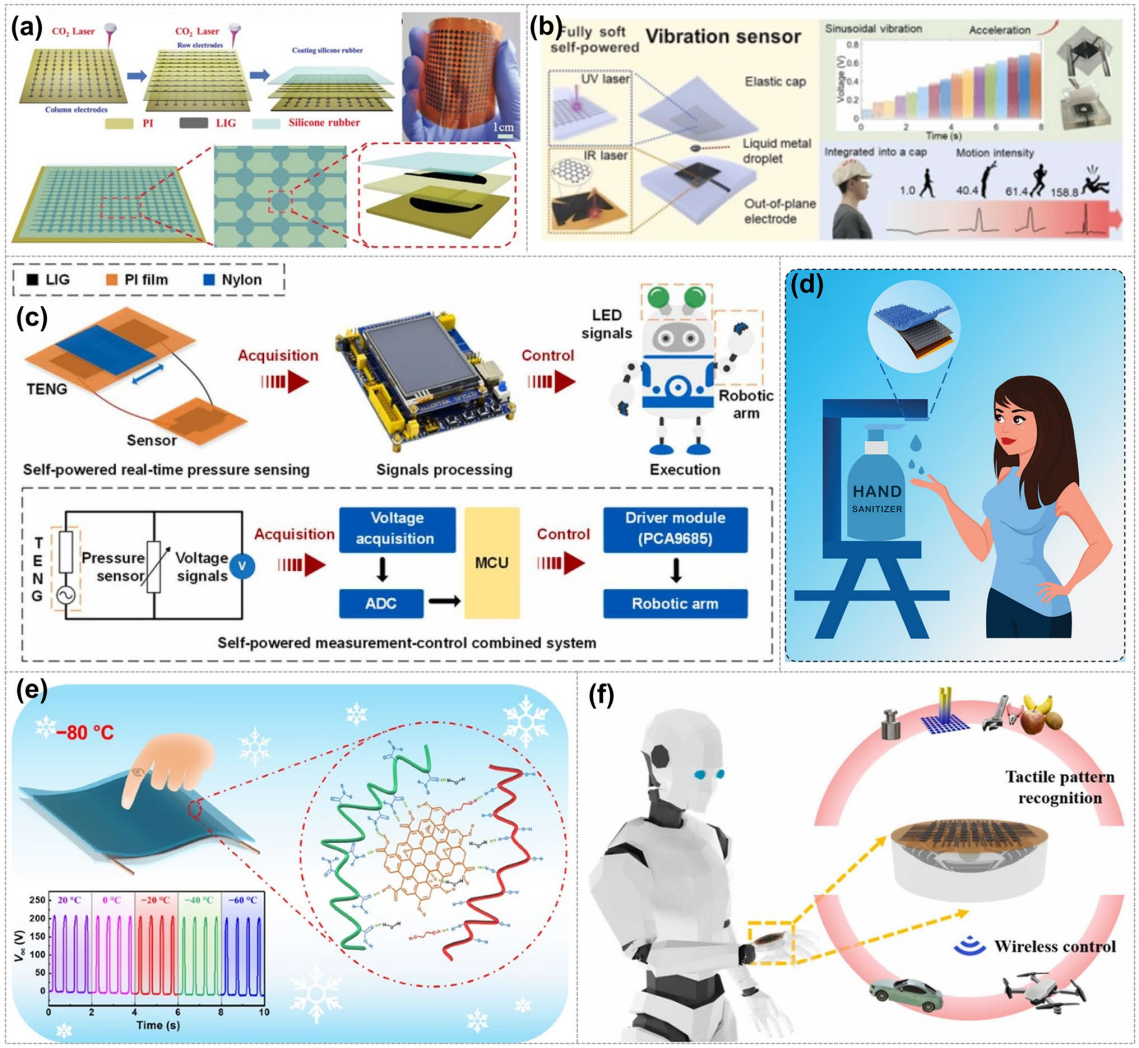
Graphene electrode-based TENG is used for physical sensors. **a** Fabrication and schematic of flexible high-resolution triboelectric sensing array based on LIG. Reproduced with permission. Reference [92] Copyright 2021, Wiley. **b** Self-powered vibration sensors can work on curved machines and track the intensity of human activities. Reproduced with permission. Reference [93] Copyright 2022, Elsevier. **c** Schematic diagrams of the self-powered measurement-control combined system. Reproduced with permission. Reference [82] Copyright 2022, Elsevier. **d** Schematic illustration demonstrating the touchless triboelectric sensor-based hand sanitizer applications. Reproduced with permission. Reference [49] Copyright 2017, Wiley. **e** Ultra-antifreeze, ultra-stretchable, transparent, and conductive hydrogel for multifunctional flexible electronics such as strain sensors and TENG. Reproduced with permission. Reference [95] Copyright 2022, Springer Nature. **f** One device achieves accurate wireless control and tactile pattern recognition of intelligent robots. Reproduced with permission. Reference [83] Copyright 2023, Elsevier

For instance, in 2021, Yan et al. constructed a smart wireless-controlled HMI system that can wirelessly control personal electronics. The system is composed of a 9-digit arrayed touch panel based on a LIG-patterned TENG (Fig. 9a) [92]. Furthermore, in 2022, Luo et al. proposed a LIG-based self-powered vibration sensor that shows its potential to operate on curved machines and track the intensity of human activities. As shown in Fig. 9b, the sensor can effectively distinguish slip from walking, jumping and running [93]. Further advancements in 2022 Xia et al. constructed a smart self-powered measurement-control combined system consisting of the LIG-based pressure sensors and TENG (Fig. 9c) [82]. Additionally, in the same year, Hu et al. developed a hydrogel-based sensor that exhibited ultra-sensitive pressure strain sensing performances. It could be utilized to recognize different utterances when attached to the surface of the throat [94]. The hydrogel-based sensor was also used as a bioelectrode to detect human electrophysiological (EP) signals. Additionally, Kumar et al. demonstrated the MoS_2_/LIG based TENG (Fig. 9d) displays outstanding humidity sensing qualities, boasting a sensitivity of 0.45 V/%. This device shows great potential for touchless interactions and minimizing potential contact scenarios [49]. Dai et al. presented a flexible and multifunctional hydrogel (GPPD-hydrogel) for use in wearable strain sensors and flexible TENG (Fig. 9e). The sensor is capable of withstanding temperatures as low as − 80 °C and demonstrates an ultra-high stretchability of up to 2,000% [95]. Lastly, in 2023, Guo et al. designed a LIG-based tactile sensing array that can achieve tactile imaging and pattern recognition. The device, shown in Fig. 9f, can be employed for accurate wireless control and real-time tactile sensing, with a pressure sensitivity of 2.2 V kPa^−1^ within the range of 0–2.8 kPa [83].

In summary, these sensors share the common feature of utilizing graphene-based advanced materials for enhanced sensing capabilities. These sensors have demonstrated applications in various fields. The integration of TENG with other materials and technologies has led to the development of smart and efficient systems for wireless control, real-time sensing, and interaction with personal electronics. Overall, the diverse applications and capabilities demonstrated in the field of TENG indicate promising directions for future research and technological advancements.

#### Graphene-Based E-Skin

Advanced technologies are increasingly needed to enable natural and efficient communication between humans and machines. Graphene-based electronic skin (e-skin) technology has emerged as a promising solution [96]. The unique properties of graphene, including its flexibility, conductivity, and transparency, have paved the way for the development of highly sensitive and responsive e-skin systems that mimic the capabilities of human skin.

**Fig. 10:**
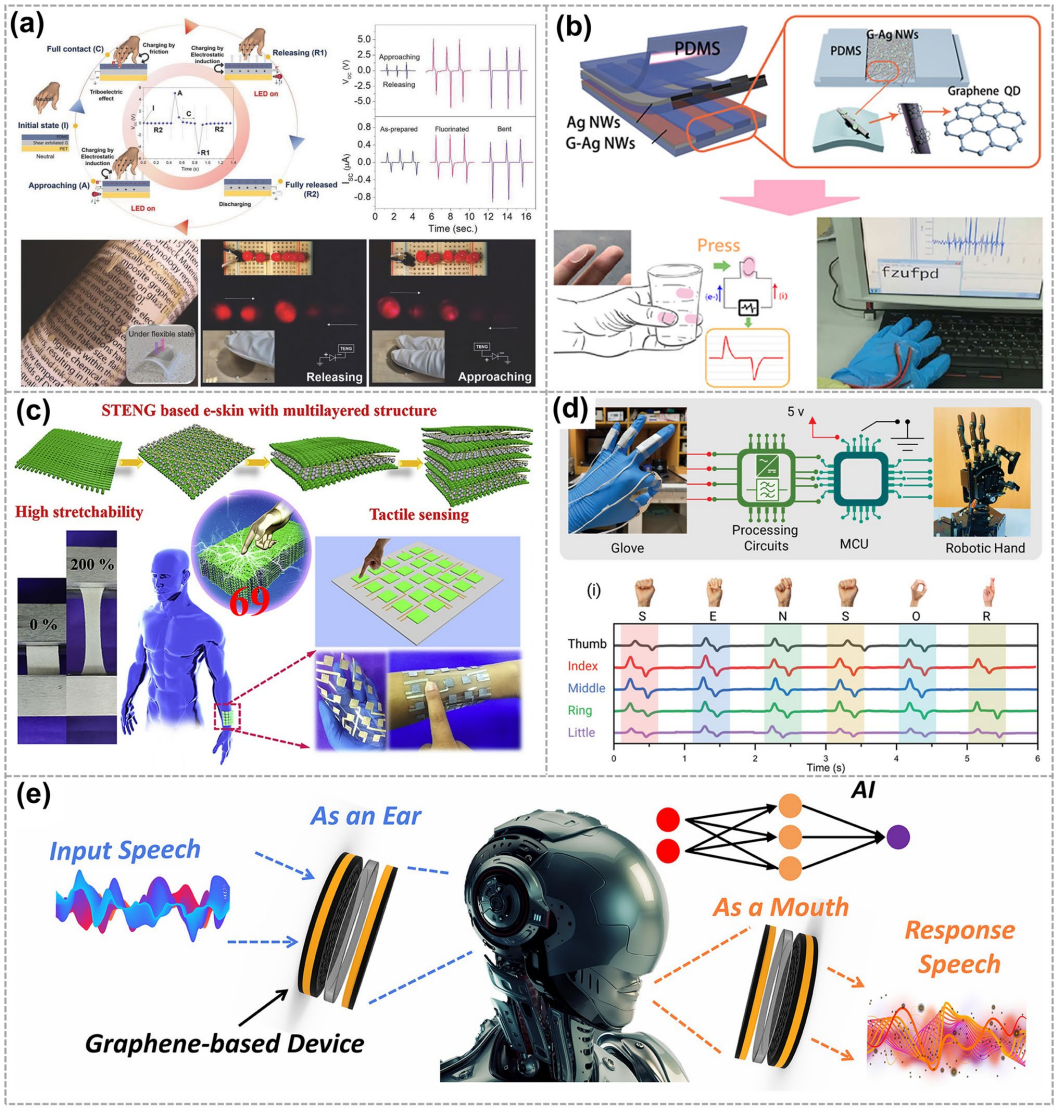
Graphene electrode-based TENG is used for e-skin. **a** Flexible and semi-transparent SE-TENG device comprising a PDMS and SEG-coated PET film by IDT. Reproduced with permission. Reference [45] Copyright 2018, Wiley. **b** Triboelectric e-skin based on graphene quantum dots for application in self-powered, smart, artificial fingers. Reproduced with permission. Reference [58] Copyright 2018, Elsevier. **c** Ultra-stretchable TENG as high-sensitive and self-powered electronic skins for energy harvesting and tactile sensing. Reproduced with permission. Reference [50] Copyright 2020, Elsevier. **d** Working principle of GHRI is applied to intelligent robots. Reproduced with permission. Reference [84] Copyright 2023, Wiley. **e** System architecture of the gesture recognition system and the system architecture of the gesture recognition system. Reproduced with permission. Reference [97] Copyright 2022, Wiley

Shin et al. fabricated a flexible and semi-transparent SE-TENG in 2018. The SE-TENG capability to generate electricity was demonstrated by connecting it to five LEDs under releasing and approaching mode (Fig. 10a) [45] and proving the potential for its application in e-skin. In the same year, Xu et al. demonstrated a cuttable, transparent, stretchable, and lightweight e-skin (Fig. 10b) [58]. By incorporating well-designed micro-gaps, this e-skin demonstrated enhanced sensitivity to various mechanical stimuli such as pressing, stretching, folding, and twisting. In 2020, Zhou et al. fabricated a flexible and self-powered electronic skin (e-skin) using an ultra-stretchable TENG. The e-skin exhibits high sensitivity (78.4 kPa^−1^) and a fast response time (1.4 ms) to pressure, showcasing its excellent tactile sensing capabilities (Fig. 10c) [50]. In 2022, Sun et al. proposed a dual-function acoustic Human–Robot Interaction (HRI) device based on graphene, known as GHRI. This device combines sound sensing (acting as an artificial ear) and emission (acting as an artificial mouth) functionalities in a single unit. By incorporating machine learning algorithms, the GHRI enables advanced speech recognition and communication capabilities, as illustrated in Fig. 10e [97]. In 2023, Kumar et al. developed a breathable triboelectric sensor based on graphene (Fig. 10d) [84]. This sensor was successfully employed in gesture recognition applications, highlighting its versatility in human–machine interfaces and soft robotics systems.

In summary, graphene-based e-skin technologies are paving the way for more intuitive and seamless human–machine interactions. With advancements such as the dual-function GHRI device, flexible and self-powered e-skin, semi-transparent SE-TENG, cuttable and stretchable e-skin, and breathable triboelectric sensor, the possibilities for enhancing communication and sensory capabilities between humans and machines are endless. These innovations not only demonstrate the potential of graphene in revolutionizing human–machine interactions but also hold promise for the future of technology and robotics.

#### Human Body Monitoring

As wearable technology continues to progress, the integration of self-powered sensors for human body monitoring is increasingly vital. The advancements in self-powered sensor technology have transformed the field of human body monitoring, allowing for real-time data collection and analysis in diverse applications [98–100].

**Fig. 11:**
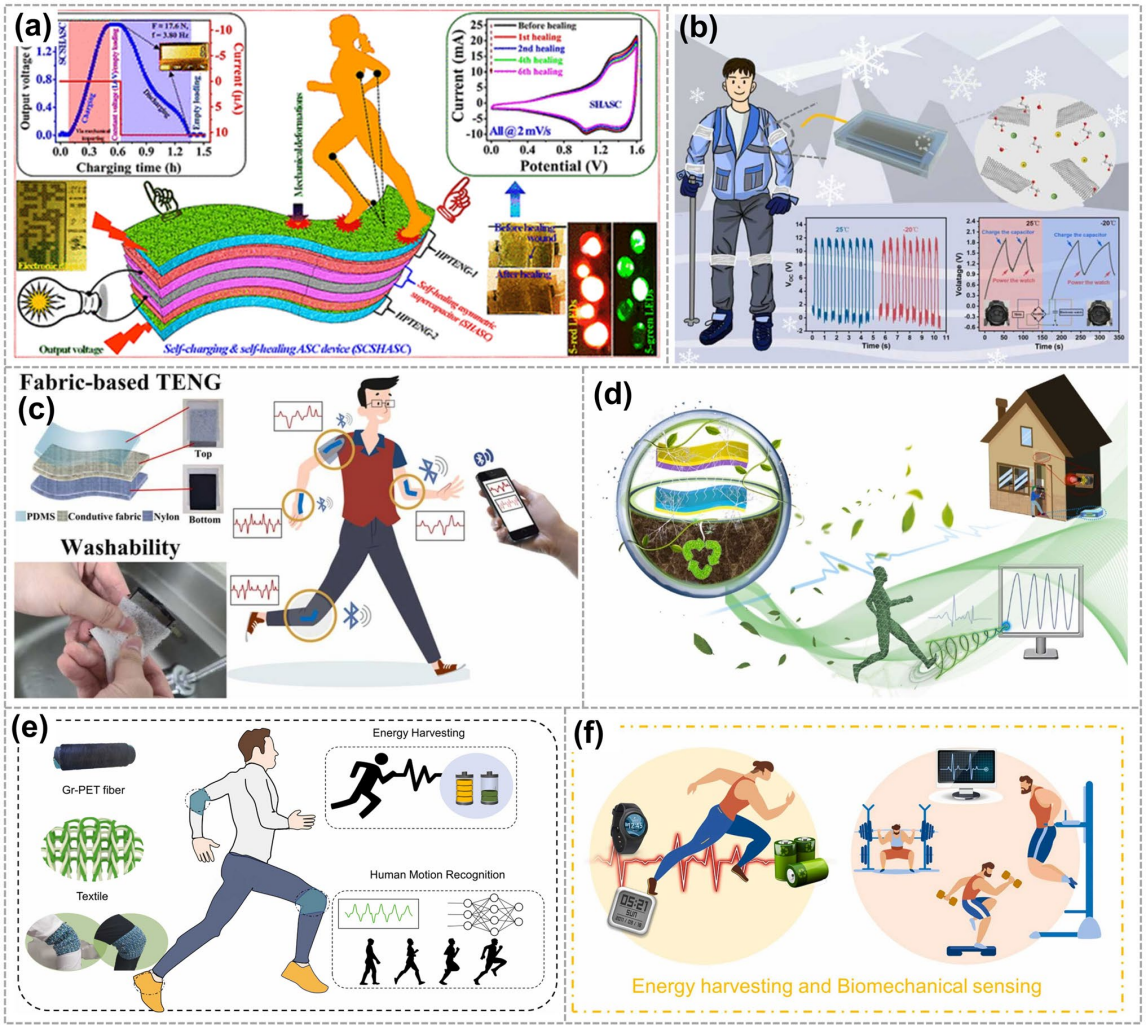
Graphene electrode-based TENG is used for human body monitoring. **a** TENG-driven self-charging and self-healing flexible asymmetric supercapacitor power cell. Reproduced with permission. Reference [101] Copyright 2019, American Chemical Society. **b** Anti-freezing and stretchable TENG based on liquid electrode for biomechanical sensing in extreme environments. Reproduced with permission. Reference [102] Copyright 2022, Elsevier. **c** TNEG was applied to four positions on the human body for sensing movement. Reproduced with permission. Reference [51] Copyright 2021, Elsevier. **d** A fully degradable TENG is used for monitoring human motions. Reproduced with permission. Reference [103] Copyright 2023, Elsevier. **e** Schematic diagram of diversified applications enabled by the 3D textile TENG. Reproduced with permission. Reference [104] Copyright 2022, Elsevier. **f** Schematic diagrams showing the application of the stretchable porous MXene/LIG foam-based TENG in human motion energy harvesting. Reproduced with permission. Reference [105] Copyright 2022, Elsevier

In 2019, Maitra et al. demonstrated a triboelectrically driven self-charging and self-healing ASC (SCSHASC) power cell (Fig. 11a). This power cell can be charged through casual body or limb movements (bio-mechanical motions) [101]. Additionally, these innovative self-powered wearable sensors, such as the liquid electrode-based TENG with graphene oxide micro/nanosheets electrode developed by Zhao et al. [102]. The device is anti-freezing and stretch-matched, making it suitable for use as a self-powered wearable sensor attached to the human body for monitoring biomechanical motion (Fig. 11b). In 2022, Yang et al. introduced a method where they combined a polydimethylsiloxane (PDMS) layer containing graphene oxide (GO) to create a fabric-based TENG. This TENG allows for the conversion of various forms of mechanical energy obtained from body motion into electronic signals, which can then be displayed on a computer (Fig. 11c) [51]. In 2023, Liu et al. proposed a fully degradable TENG with graphene composite paper as electrodes. It could charge up different electronic devices, monitoring human motions (Fig. 11d). The TENGs offer the capability to monitor biomechanical motion and human movements with high accuracy and efficiency [103]. In the same year, Xiong et al. developed a consecutive and scalable process for graphene textile TENGs (Fig. 11e) [104]. These TENGs have demonstrated high flexibility, shape adaptability, and structural integrity, making them suitable for energy harvesting and human motion recognition. Yang et al. reported on an intrinsically stretchable TENG that integrates Ag NWs/LIG electrodes with triboelectric MXene/PDMS-Ecoflex composites (Fig. 11f) [105]. This innovation aims to achieve efficient mechanical energy harvesting and self-powered biomechanical sensing.

In summary, the evolution of self-powered sensors for monitoring the human body is revolutionizing the wearable technology field. These devices, such as the self-power TENGs with graphene electrodes, enable real-time data collection and analysis in various applications, including biomechanical sensing. With advancements in this technology, monitoring biomechanical motion and human movements with great accuracy and efficiency has become achievable, paving the way for enhanced healthcare, sports performance, and overall well-being.

#### Alarm System

Graphene electrode-based TENG has emerged as a key technology in the development of advanced alarm systems. These TENG devices offer high sensitivity and self-powering capabilities, enabling the design of innovative alarm systems that can effectively monitor and detect potential dangers.

**Fig. 12:**
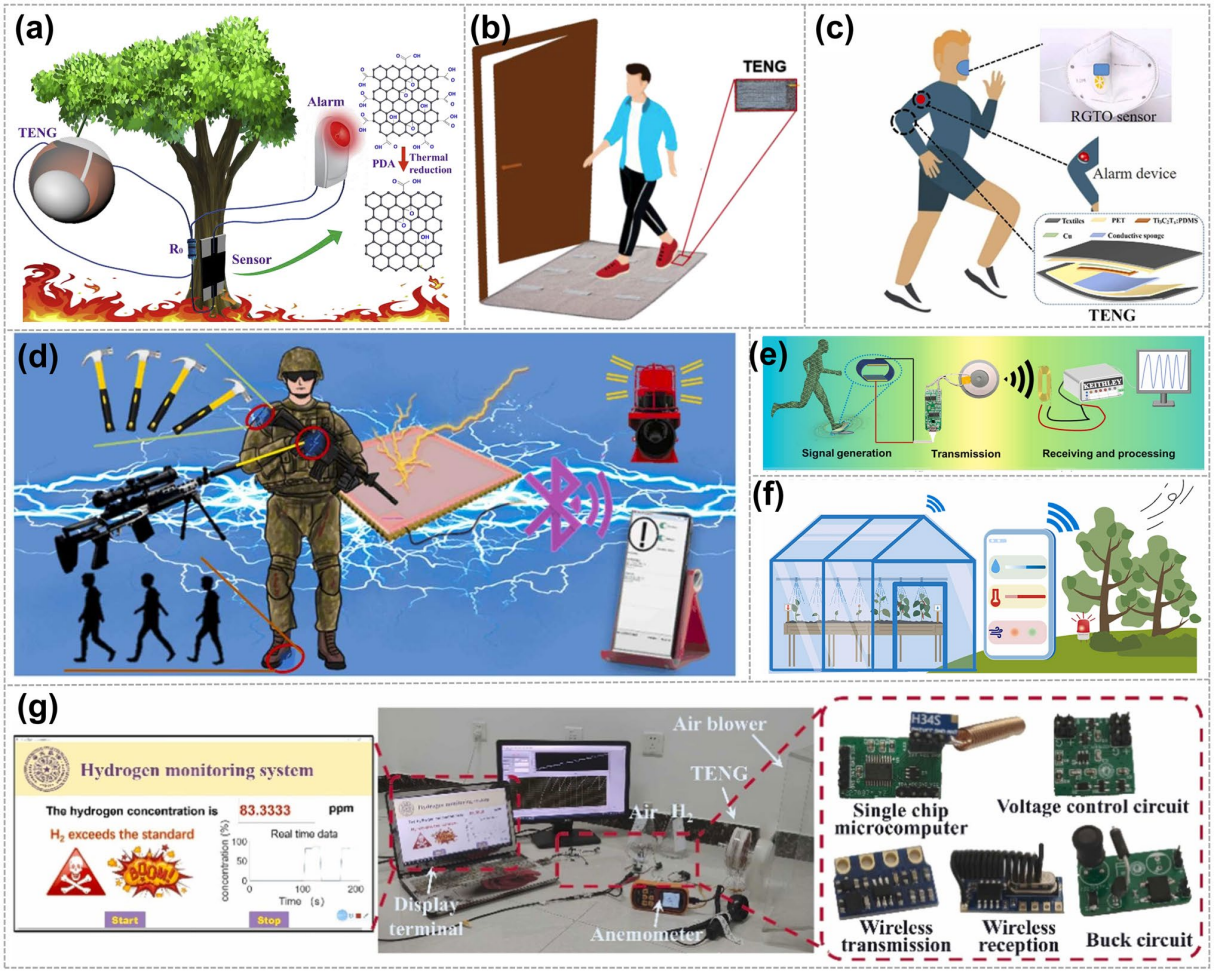
Graphene electrode-based TENG is used in alarm systems. **a** Schematic illustration of self-powered forest fire alarm system. Reproduced with permission. Reference [52] Copyright 2020, Elsevier. **b** Schematic diagram of carpet signal monitor work. Reproduced with permission. Reference [106] Copyright 2020, Elsevier. **c** Respiration monitoring alarm device based on TENG-RGTO-5 sensor. Reproduced with permission. Reference [107] Copyright 2020, Elsevier. **d** Enhanced Kevlar-based TENG with anti-impact and sensing performance toward wireless alarm system. Reproduced with permission. Reference [108] Copyright 2021, Elsevier. **e** Schematic diagram of the wireless sensing for human movements. Reproduced with permission. Reference [103] Copyright 2023, Elsevier. **f** Potential applications of the LPL-TENG in smart agriculture. Reproduced with permission. Reference [109] Copyright 2022, Elsevier. **g** Real-time monitoring and hydrogen leakage alarm system. Reproduced with permission. Reference [53] Copyright 2023, Elsevier

In 2020, Liu et al. demonstrated a self-powered forest fire alarm system (Fig. 12a). The self-powered FFAS based on TENG achieves a low-temperature response (160 °C), the rapid response time (~ 3 s), and especially no external power source supply for early forest fire monitoring and detection [52]. Furthermore, in the same year, He et al. designed a safety warning device that can monitor carpet signals using a GO-based TENG. When a person walks by, their sole contacts and separates from the fabric, generating a signal that is then transmitted to the programming system (Fig. 12b) [106]. In 2022, Yu et al. designed a breathing alarm device for humans (Fig. 12c) [107]. The device utilized an rGO-TiO_2_ humidity sensor as the respiratory sensing component, which features high responsiveness, fast response and recovery times, high reversibility, minimal hysteresis, and excellent stability. Additionally, in 2022, Wang et al. developed a graphene-based TENG with anti-impact and sensing performance for wireless alarm systems. The system, shown in Fig. 12d, designed with high sensitivity, is intended to monitor and alert users to potential impact dangers, thereby paving the way for the advancement of next-generation intelligent protective clothing [108]. Moreover, in 2023, Liu et al. developed a burglar alarm that can be placed under the doormat when the homeowner leaves the house. Should any strangers attempt to enter the house and step on the doormat, the FD-TENG sensor will be triggered and generate a voltage signal, activating the buzzer and LED lamp in the alarm system (Fig. 12e) [103]. Recently, Luo et al. proposed a living plant leaf-based TENG(LPL-TENG) for self-powered smart agriculture sensing (Fig. 12f), the feature of alarming at high wind speed can also be exhibited by the LPL-TENG to realize remote control [109]. In the same year, Wang et al. designed a hydrogen leakage alarm to ensure the safety and stability of the hydrogen production process. At a wind speed of 4.5 m s^−1^, the wind-driven TENG consistently powered real-time monitoring, wireless transmission, and hydrogen leakage alarm (Fig. 12g) [53].

In summary, graphene electrode-based TENG have transformed the landscape of alarm systems with their high sensitivity and self-powering capabilities. These innovative devices have been successfully incorporated into a variety of applications, including forest fire detection, impact sensing in protective suits, human respiration monitoring, and smart agriculture, enhancing safety measures in all aspects of life and enabling real-time monitoring of potential hazards. As research and development in TENG technology continue to advance, the future of alarm systems looks promising, offering increased safety and security in a wide range of scenarios.

### Performance Improvement of TENG

As research on TENG continues to intensify, it becomes crucial to explore strategies to enhance the performance of TENGs. The studies mentioned in the following text shed light on the successful integration of graphene and its derivatives in TENG devices, demonstrating their potential to significantly improve power output and efficiency. By understanding these advancements, we can grasp the importance of these integrations and their implications for the further development and application of TENG technology.

As shown in Table 1, Zhang et al. embedded graphene sheets in carbon for TENG electrodes, resulting in higher electric output performance compared to the original TENG. The peak power density increased from 0.5 to 0.63 mW cm^−2^, raising efficiency by 20% [110]. According to Fatemeh et al., the incorporation of GO within micro-patterned PDMS at 1 wt% (PDMS@1CGO) significantly enhanced the triboelectric characteristics. The output performance of the micro-patterned film was improved by 46% [111]. Chen et al. developed a stretchable TENG based on stretchable crumpled graphene that enhances performance. The power density is 0.25 mW cm^−2^, which is 20 times higher than that of a planar graphene-based TENG [112]. Chen et al. demonstrated a simple and effective method to enhance the performance of the flexible TENG based on AuCl_3_-doped crumpled graphene (CG). The output voltage and current of the TENG showed a 5 times enhancement with the doping concentration compared to the CG-based device [113]. Liu et al. utilized graphene composite paper to replace copper electrodes, resulting in improved output performance. The optimal power density reached 675 mW m^−2^, which was approximately 5.05 times higher than that of the TENG employing copper electrodes [103]. Shi et al. fabricated high-performance TENGs by incorporating graphene nanosheets using electrospinning technology. The resulting TENGs exhibited a maximum peak power density of 130.2 W m^−2^, which was nearly eight times higher compared to the original TENG [44]. Huang et al. modified the surface electrification of the triboelectric materials and enhanced the output performance of the TENG by embedding GO in the PVDF matrix. As a result, the power density experienced a remarkable increase of 513% [114]. Viyada et al. achieved an enhanced power output of a TENG by utilizing GO and sodium dodecyl sulfate to modify PDMS. The resulting power output values were threefold higher than those of the flat PDMS configuration [46]. Kinas et al. significantly enhanced the output voltage of the TENG by grafting reduced graphene oxide (rGO) and carbon nanotubes (CNT) onto polyacrylonitrile (PAN). This modification resulted in a remarkable increase of 125% in the maximum peak power density [115]. Hatta et al. prepared a mixture of PDMS/BTO doped with GQDs and mechanically stirred it. The resulting TENGs exhibited an output performance that was 2 times higher compared to nanocomposite films without GQDs [59]. Choi et al. found that the optimal amount of GQD incorporation stimulated the formation of the polar β-phase and enhanced the performance of TENG. The maximum output power from TENG devices increased from 35 to 97 μW [61].

**Table 1:** Enhancement of TENG performance by various types of graphene electrodes

Graphene electrode types	Enhanced power density (mW cm^−2^)	Increased efficiency (%)	Refs
Graphene sheets	0.63	26	[110]
GO nanoparticles	0.75	46	[111]
Crumpled graphene	0.25	2000	[112]
Crumpled graphene	0.032	500	[113]
Graphene-paper	0.0675	505	[103]
Graphene nanosheets	13.02	800	[44]
GO	0.23	513	[114]
GO	4.8	300	[46]
rGO	1.4	125	[115]
GQDs	0.16	200	[59]
GQDs	0.0027	277	[61]

In summary, the incorporation of graphene and its derivatives in TENG devices has consistently demonstrated significant improvements in power output and performance compared to the original TENG. These improvements are important for the efficiency and performance enhancement of TENG and provide strong support for the further development and application of TENG technology.

### Other Applications

These innovative applications of graphene electrode-based TENG demonstrate the diverse range of possibilities in various fields. Researchers are continuously exploring new ways to utilize graphene in unique and breakthrough applications, demonstrating the potential for further advancements in renewable energy and environmental protection.

**Fig. 13:**
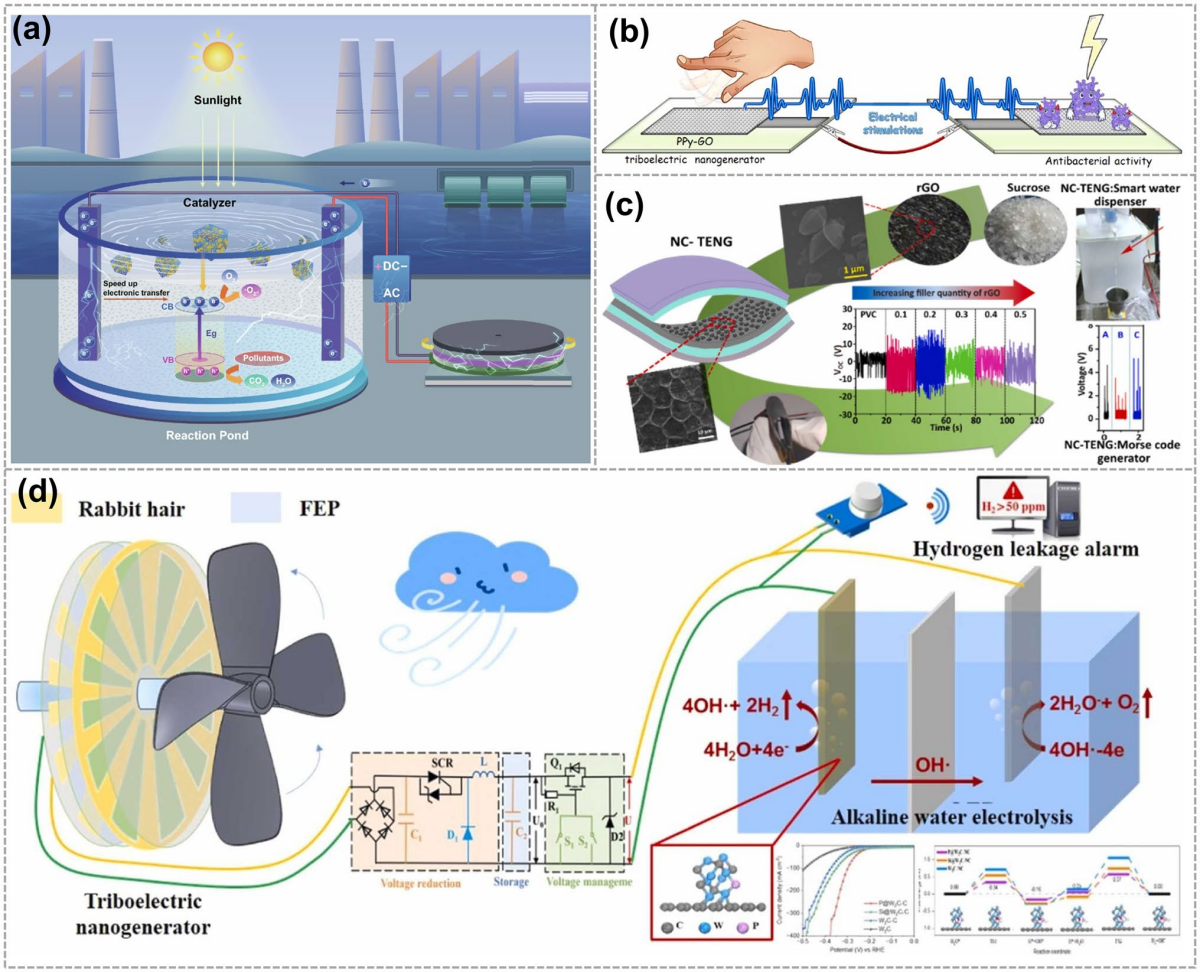
Graphene electrode-based TENG is used for other applications. **a** Schematic illustration of high-efficiency wastewater purification system based on TENG. Reproduced with permission. Reference [116] Copyright 2021, Springer Nature. **b** TENG-induced electrical stimulations resulted in the rupture of bacteria membranes. Reproduced with permission. Reference [117] Copyright 2023, Elsevier. **c** NC-TENG is demonstrated as a humidity sensor, Morse code generator, and smart water dispenser. Reproduced with permission. Reference [118] Copyright 2022, Elsevier. **d** P-doped W_2_C nanoparticles for hydrogen evolution reaction powered by a wind-driven TENG. Reproduced with permission. Reference [53] Copyright 2022, Elsevier

In 2021, Shen et al. developed a distinctive hybrid system by combining a self-powered TENG with a three-dimensional graphene oxide photocatalyst doped with carbon dots-TiO_2_ sheets (Fig. 13a). The power generated by the TENG can be used directly for wastewater purification, showcasing a self-powered electrocatalytic technology [116]. In 2023, Marziyeh et al. created a composite TENG consisting of polypyrrole-graphene oxide (PPy-GO) with enhanced electrical outputs and bactericidal properties (Fig. 13b). The findings illustrate that the combination of GO nanosheets and TENG-generated electrical stimuli had a synergistic effect, leading to enhanced ROS generation and the rupture of the cellular membrane in *S. aureus* as the bacterial model [117]. In 2023, Rumana et al. developed an affordable and innovative nanocomposite-based TENG (NC-TENG) utilizing reduced graphene oxide (rGO). The NC-TENG (Fig. 13c) is showcased as a humidity sensor, Morse code generator, and smart water dispenser [118]. 2024 Wang et al. demonstrated P-doped W_2_C nanoparticles anchored on graphene (P@W_2_C-C) as a highly effective electrocatalyst for the hydrogen evolution reaction, powered by a wind-driven TENG (Fig. 13d). They successfully achieved a hydrogen production rate of 64.5 μL min^−1^ and implemented a self-awakening hydrogen leakage alarm system [53].

As described earlier, there exists some graphene electrode-based TENGs that can drive miniature commercial electronic devices [109, 118]. However, achieving wide-scale commercialization involves several challenges as follows. Firstly, consistency and stability: while graphene electrode-based TENGs perform well in small-scale experiments, it is crucial to ensure the technology's consistency and stability under various environmental conditions in commercial applications. Secondly, standardization: the standardization of technology can impact its adaptability and scalability across different application scenarios. Developing and adhering to relevant industry standards can facilitate better market integration. Finally, demand and acceptance: the commercialization of the technology requires careful consideration of market demand and acceptance, necessitating thorough market research. Therefore, the primary challenge for graphene electrode-based TENGs in commercial applications lies in validating performance beyond lab-scale environments and addressing issues such as cost, reliability, and integration.

In summary, the continuous exploration and development of graphene electrode-based TENG highlight the immense potential for further innovation in the fields of renewable energy and environmental protection. These advancements underscore the importance of harnessing the unique properties of graphene to drive sustainable solutions and pave the way for a greener, more efficient future and commercial applications.

## Conclusions and Prospects

This review outlines the significant developments in graphene electrode-based TENG research that have been made in recent years. As a naturally self-powered device, TENG can effectively address the issue of energy supply for microdevices such as wearable electronics. Graphene and its derivatives were processed as electrodes for TENG by both top-down and bottom-up methods, which resulted in a notable enhancement in power output and performance. The exceptional electrical conductivity, mechanical strength, and dopability of graphene facilitate the enhancement of TENG efficiency and reliability in energy harvesting. Its excellent flexibility, conductivity, and transparency can be employed to develop highly sensitive and responsive e-skins, as well as to enable highly accurate and efficient monitoring of human motion and sensing. Furthermore, graphene electrode-based TENG technology has the potential to contribute to the development of renewable energy and environmental protection, including the production of hydrogen and the purification of water.

With the continuous expansion of graphene‘s use in TENGs, future research will focus on developing graphene with enhanced properties. Consequently, technologies for processing high-performance graphene are constantly evolving, including doping, surface functionalization, and other methods. For example, doping with nitrogen, boron, or transition metals can significantly enhance its electrical conductivity and catalytic activity, thus improving the energy harvesting efficiency of TENGs. Additionally, functionalization technologies, which involve adding chemical groups to graphene's surface, can customize its properties for specific applications and enhance the stability of the electrode, ultimately increasing the robustness of the TENG system. Therefore, advancements in graphene processing technology are driving TENGs toward becoming self-powered wearable sensing systems with high energy harvesting efficiency, long life, and high stability. In addition, exploring alternative materials to graphene for TENG electrodes, such as 2D materials like MXenes, is also a promising research direction.

To advance the development of graphene-based TENGs, several challenges must be addressed. For example, using naturally degradable materials (e.g., cork and fruit shells) as carbon precursors in LIG and enabling precise pattern customization can improve material utilization efficiency and reduce waste generation, thereby achieving environmentally friendly processing. To ensure processing consistency, errors can be minimized by improving the stability of the laser light source and increasing the precision of the processing platform. Additionally, pre-treating raw materials to reduce defects is beneficial. Furthermore, gaining a deeper understanding of the charge transfer mechanisms between graphene-based TENG electrodes and modifying surface morphology to increase electrode contact area are essential for improving energy harvesting efficiency. The robustness of the system can be improved by targeted functionalization of the electrode surface to increase the long-term life cycle and high stability of the system.

**Fig. 14:**
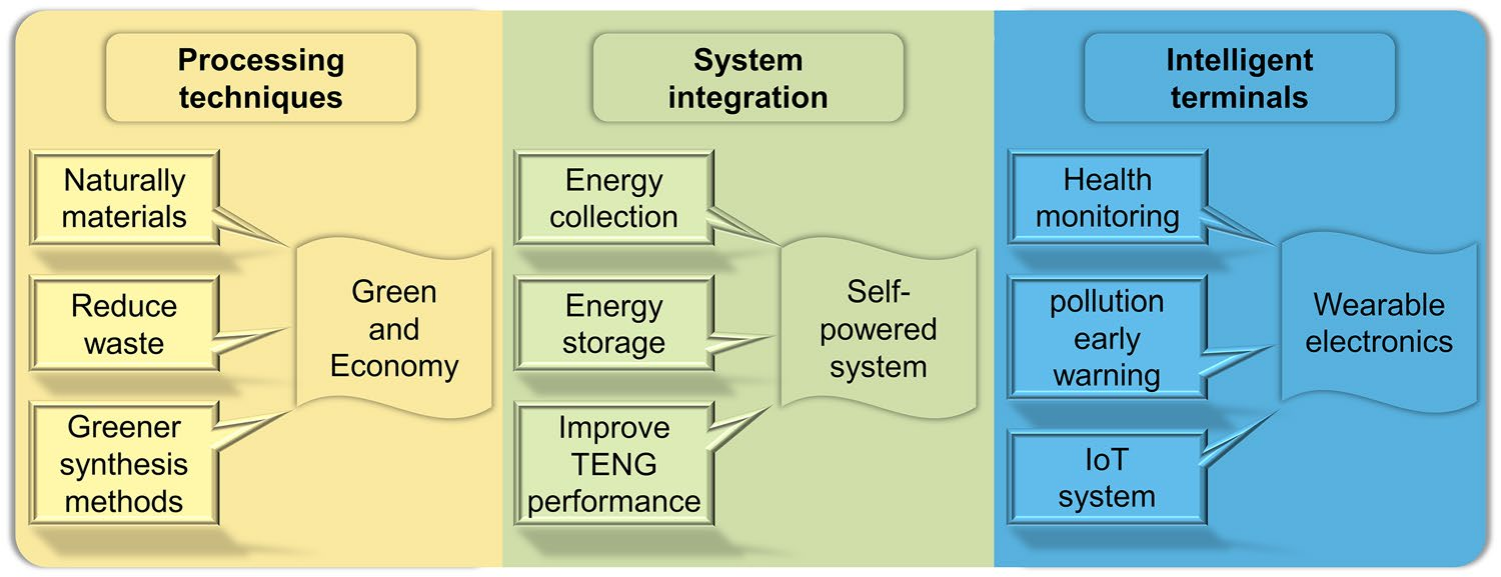
Prospects for the development of graphene electrode-based TENG

As the field of graphene electrode electrode-based TENG technology continues to evolve, self-powered graphene electrode-based TENGs are poised to become a valuable addition to the technology landscape. This review proposes the following outlooks for future research and practical applications (Fig. 14).

1. **Processing techniques**: The majority of the previously mentioned top-down methods incorporate graphene and its derivatives into polymers such as PDMS and PVDF to form composite materials, which are used as electrodes for TENGs. Although these methods can enhance the performance of TENG, the synthesis process of electrode materials is arduous, and some of the methods present safety hazards and generate waste materials. Among the bottom-up methods, the CVD method has high equipment maintenance costs. Furthermore, the LIG processing method is prone to defects in the graphene. Developing greener synthesis methods is crucial for improving environmental sustainability in graphene production. Consequently, future research may be directed toward the development of an environmentally friendly and cost-effective electrode fabrication method based on natural materials. Future strategies might focus on optimizing production techniques and enhancing material utilization efficiency to ensure the environmental sustainability of graphene production.
2. **System integration**: TENG is a device that converts the energy of the surrounding environment into electricity. The peak power density of graphene electrode-based TENG is close to hundreds of watts per square meter, which is fully capable of storing and powering electronic devices. Kumar et al. integrated TENG and supercapacitor into a single device, thereby achieving system integration of energy harvesting and storage. This was applied to a self-powered smart switching system [119]. In light of the potential applications of graphene electrode-based TENGs in self-powered systems, future research may be directed toward the development of TENGs with high-performance energy storage systems.
3. **Intelligent terminals**: The issues of human health and environmental protection remain at the forefront of public concern. The potential of graphene electrode-based TENG has already been demonstrated in a number of applications, including human movement monitoring, wastewater purification, hydrogen production and sterilization. The integration of TENG-based self-powered systems with the Internet of Things (IoT) will result in the development of smart terminal systems for the real-time monitoring of human health and environmental energy. This will facilitate the early detection of potential issues and enable the implementation of corrective measures.
